# Left atrial pressure and significant tricuspid regurgitation in persistent atrial fibrillation

**DOI:** 10.3389/fcvm.2025.1575750

**Published:** 2025-06-06

**Authors:** Yuewu Lin, Maolin Ye, Yan Qiu, Dawei Lin, Sezhang Ke

**Affiliations:** ^1^ Zhangzhou Affiliated Hospital of Fujian Medical University, Department of Geriatrics, Zhangzhou, Fujian, China; ^2^ Department of Cardiology, Zhongshan Hospital, Fudan University, Shanghai, China

**Keywords:** tricuspid regurgitation, left atrial pressure, atrial fibrillation, right heart remodeling, left atrial dysfunction

## Abstract

**Introduction:**

Atrial fibrillation (AF) is a well-established contributing factor to isolated tricuspid regurgitation (TR), with elevated left atrial pressure (LAP) playing a crucial role in disease progression and patient outcomes. We investigated the relationship between LAP and TR in patients with AF.

**Methods:**

We enrolled individuals diagnosed with AF who underwent LA appendage closure at two centers in China, between January 2015 and December 2023. Participants were classified into two groups based on TR severity: those with moderate-to-severe TR and those with no significant TR groups. Baseline characteristics, imaging findings, and follow-up data were analyzed.

**Results:**

A total of 189 participants were included, of whom 60 had moderate-to-severe TR. Compared to the no-TR group, the moderate-to-severe TR group was older (74.22 ± 9.71 years vs. 69.37 ± 8.04 years, *p* < 0.001), had a longer history of persistent AF (7.41 ± 7.18 years vs. 2.08 ± 2.26 years, *p* < 0.001), and exhibited lower hemoglobin and hematocrit levels. In addition, patients in the moderate-to-significant TR group were more likely to have mitral regurgitation, larger LA diameters, higher LA systolic pressure (LASP), higher LA diastolic pressure, higher mean LA pressure, and pulmonary hypertension (all *p* < 0.001). Elevated LAP was strongly associated with right heart remodeling and significant TR in patients with persistent AF. Regression analysis identified LASP, mitral regurgitation, and AF duration as independent predictors of significant TR (all *p* < 0.01).

**Conclusions:**

Early identification of LAP elevation and right heart remodeling may guide targeted interventions to prevent TR progression and improve patient outcomes. Furthermore, the recognition of LASP and AF duration as predictors of TR and LA dysfunction emphasizes the need for thorough clinical assessments in treatment planning.

## 1 Introduction

Secondary or functional tricuspid regurgitation (TR) is caused by annular dilation and leaflet maladaptation, primarily driven by right heart remodeling. This condition often arises from left-sided heart failure, particularly in the context of mitral or aortic valvulopathy (1, 2). Recent studies highlight atrial fibrillation (AF) as a major risk factor for isolated or idiopathic TR when no other structural abnormalities are present (3, 4). The underlying mechanism involves right atrial (RA) enlargement and tricuspid valve annular dilation induced by arrhythmia.

Patients with AF often present with elevated left atrial pressure (LAP) and stiffness, which are linked to chronic atrial stretch, resulting in atrial dilatation, fibrosis, and loss of contractile elements (5, 6). Conversely, elevated LAP and volume overload predispose individuals to AF, serving as independent predictors of AF recurrence following pulmonary vein isolation (7). Importantly, LAP acts as a critical bridge between left-sided heart dysfunction and right-heart remodeling. Persistently elevated LAP contributes to atrial remodeling, including RA dilation and tricuspid annular enlargement, and may also lead to RV dilation. RV remodeling can further impair tricuspid leaflet coaptation, and in many patients, TR reflects a combination of atrial and ventricular mechanisms. This hemodynamic interplay underscores the importance of evaluating LAP in AF-related TR to improve patient management. Notably, increased LAP may also contribute to the development of TR in patients with AF.

Transcatheter treatment for TR has become a critical option for managing severe TR in patients at high risk for surgical complications (8). However, hemodynamic shifts following TR repair or replacement may result in right ventricular overload. Elevated left ventricular diastolic pressure can increase LAP, leading to pulmonary venous congestion and pulmonary hypertension, which may eventually contribute to right atrial pressure overload and right heart remodeling. For patients with AF, elevated LAP and impaired diastolic function of the left ventricle may limit the success of these interventions. To address this, we sought to explore the association between LAP and TR in patients with AF.

## 2 Materials and methods

### 2.1 Research participants

This retrospective study included patients with persistent AF who underwent LA appendage closure at Zhangzhou Affiliated Hospital of Fujian Medical University (Fujian, P.R China) and Zhongshan Hospital Fudan University (Shanghai, P.R China), between January 2015 and December 2023. All participants met the eligibility criteria for LA appendage closure (CHA_2_DS_2_-VASc score ≥2 and for whom oral anticoagulation therapy was deemed unsuitable). The exclusion criteria were congenital heart disease, prior tricuspid valve surgery, primary TR, hemodialysis or peritoneal dialysis, history of heart transplantation, reliance on a ventricular assist device, repeated diagnostic assessments, poor-quality echocardiographic images, and paroxysmal AF. To minimize the potential impact of device-related TR, patients with implanted cardiac devices (e.g., pacemakers) were not included in this study. The AF duration was confirmed using electrocardiograms or medical records, with persistent AF defined as lasting more than 7 days.

### 2.2 Patient classification and data collection

Baseline characteristics and clinical data were obtained from electronic health records and the catheterization laboratory database for retrospective analysis. Transthoracic echocardiography was performed using the Vivid 7GE system (GE Healthcare, Chicago, IL, USA) by an experienced sonographer. The LA diameter was measured in the anteroposterior plane using the parasternal long-axis view, while other key echocardiographic parameters were assessed according to standard echocardiographic guidelines. Key echocardiographic parameters, such as the RA diameter and tricuspid annular (TA) plane systolic excursion, were validated by two independent cardiologists. TR and MR severities were both graded based on the valve regurgitant jet area observed during echocardiographic assessments. TR was categorized into six grades: grade 0 (no or trace regurgitation), grade 1 (mild), grade 2 (mild-to-moderate), grade 3 (moderate), grade 4 (moderate-to-severe), and grade 5 (severe). MR was classified similarly into five grades (0–4) based on current echocardiographic recommendations. This study adhered to the principles outlined in the Declaration of Helsinki and was approved by the relevant ethics committee. Written informed consent was obtained from all patients.

### 2.3 Right heart catheterization

Right heart catheterization was performed by experienced cardiologists to assess LAP and pulmonary artery pressure. Under sterile conditions and local anesthesia, a multipurpose catheter was inserted into the right atrium via the inferior vena cava and advanced into the pulmonary veins. The LAP was directly measured using transseptal puncture during LA appendage closure, allowing for direct pressure recording within the left atrium rather than relying on pulmonary wedge pressure estimation. The LAP, including the LA systolic pressure (LASP; defined as the v-wave peak) and left atrial diastolic pressure (LADP; measured immediately following the y-descent), was measured at end-expiration, and the mean LAP (mLAP) was calculated. In patients with AF, the averages from three consecutive cardiac cycles were used to ensure hemodynamic stability. A Swan–Ganz balloon-tipped catheter was also introduced through the right atrium and ventricle into the pulmonary artery. Hemodynamic parameters, including LASP, LADP, mLAP, pulmonary artery systolic pressure (PASP), and mean pulmonary artery pressure (mPAP), were recorded during the procedure.

### 2.4 Statistical analysis

Patients were categorized into two groups based on TR severity, assessed using transthoracic echocardiography. For analysis, patients with grades 0–2 were placed into the “without significant TR” group, and those with grades 3–5 into the “moderate-to-severe TR” group. Continuous variables with normal distributions are presented as mean ± standard deviation, whereas categorical variables are presented as percentages. The unpaired Student's *t*-test was used to evaluate differences between the two groups, and the chi-squared test was applied for categorical data. Univariate and multivariate logistic regression analyses were performed to evaluate the strengths of associations. A *p*-value < 0.05 was considerced statistically significant. All statistical analyses were performed using Stata 15.1 (StataCorp LLC, College Station, TX, USA) or SPSS 26.0 (IBM Corp., Armonk, NY, USA) software.

## 3 Results

### 3.1 Baseline clinical and imaging characteristics

This study included 189 patients, and 60 presented with moderate-to-severe TR. Compared with patients with AF without significant TR, those with moderate-to-severe TR had a higher average age and longer average AF duration. These patients also exhibited lower hemoglobin levels (127.94 ± 17.66 g/L vs. 136.98 ± 16.28 g/L, *p* = 0.002) and hematocrit (38.25 ± 4.82% vs. 40.59 ± 4.58%, *p* = 0.004), but higher serum B-type natriuretic peptide concentrations (486.65 ± 858.94 pg/mL vs. 132.97 ± 251.54 pg/mL, *p* < 0.001) (Table 1).

**Table 1:** Comparison of baseline characteristics and blood parameters between the two groups.

Patient characteristics	Without moderate-to-severe TR group (*n* = 129)	Moderate-to-severe TR group (*n* = 60)	*p*-value
Age, years	69.37 ± 8.04	74.22 ± 9.71	<0.001
Male	83 (64.3%)	35 (58.3%)	0.43
Hypertension	78 (60.5%)	35 (58.3%)	0.78
Diabetes mellitus	14 (10.9%)	11 (18.3%)	0.16
Coronary artery disease	24 (18.6%)	9 (15.0%)	0.54
Heart failure	11 (8.5%)	11 (18.3%)	0.050
Stroke	40 (31.0%)	16 (26.7%)	0.54
AF duration, years	2.08 ± 2.26	7.41 ± 7.18	<0.001
Hemoglobin, g/L	136.98 ± 16.28	127.94 ± 17.66	0.002
Hematocrit, %	40.59 ± 4.58	38.25 ± 4.82	0.004
BNP, pg/mL	132.97 ± 251.54	486.65 ± 858.94	<0.001

Abbreviations: AF, atrial fibrillation; BNP, brain natriuretic peptide; TR, tricuspid regurgitation.

### 3.3 Imaging findings

As shown in Table 2, patients with moderate-to-severe TR were more likely to have severe mitral regurgitation (*p* < 0.001) and larger LA diameters (49.75 ± 6.10 vs. 46.96 ± 5.80, *p* = 0.003) than patients without significant TR. Right heart remodeling was evident in patients with moderate-to-severe TR, as indicated by a higher RA area (RAA; 35.60 ± 9.06 cm^2^ vs. 21.68 ± 4.84 cm^2^, *p* < 0.001) and right ventricular end-systolic area (12.63 ± 4.14 cm^2^ vs. 10.60 ± 2.89 cm^2^, *p* < 0.001) than patients without significant TR. Additionally, significantly lower TA plane systolic excursion (16.08 ± 3.20 mm vs. 17.52 ± 3.41 mm, *p* = 0.007) in patients with moderate-to-severe TR suggested impaired right ventricular systolic function. Right heart catheterization confirmed that moderate-to-severe TR correlated with elevated LASP, LADP, mLAP, PASP, and mPAP (all *p* < 0.001).

**Table 2:** Analysis of baseline imaging characteristics in the two groups.

Parameters	Without significant TR group (*n* = 129)	Moderate-to-severe TR group (*n* = 60)	*p*-value
Echocardiography			
LAD (mm)	46.96 ± 5.80	49.75 ± 6.10	0.003
LVEDd (mm)	48.92 ± 5.10	47.95 ± 5.94	0.25
LVEDs (mm)	32.18 ± 4.77	31.27 ± 4.31	0.21
LVEF, %	62.43 ± 6.49	63.38 ± 4.58	0.31
MR degree			
0	62 (49.6%)	9 (15.0%)	<0.001
1	49 (39.2%)	28 (46.7%)	
2	11 (8.8%)	11 (18.3%)	
3	3 (2.4%)	10 (16.7%)	
4	0 (0.0%)	2 (3.3%)	
TA diameter, mm	29.34 ± 5.64	35.45 ± 4.57	<0.001
RAA, cm^2^	21.68 ± 4.84	35.60 ± 9.06	<0.001
RVESA, cm^2^	10.60 ± 2.89	12.63 ± 4.14	<0.001
TAPSE, mm	17.52 ± 3.41	16.08 ± 3.20	0.007
Right heart catheterization			
LASP, mmHg	18.31 ± 5.80	36.80 ± 8.74	<0.001
LADP, mmHg	2.90 ± 3.74	6.47 ± 5.41	<0.001
mLAP, mmHg	9.75 ± 4.01	18.25 ± 5.91	<0.001
PASP, mmHg	31.81 ± 6.79	49.47 ± 13.15	<0.001
PADP, mmHg	8.02 ± 4.66	12.98 ± 7.01	<0.001
mPAP, mmHg	17.91 ± 4.85	27.33 ± 9.11	<0.001
mPAP ≥20 mmHg	39/129 (30.2%)	50/60 (83.3%)	<0.001

Abbreviations: LVEF, left ventricular ejection fraction; LVEDd, left ventricle end-diastolic dimension; LVEDs, left ventricle end-systolic dimension; LAD, left atrial diameter; MR, mitral regurgitation; TA, tricuspid annulus; RAA, right atrial area; RVESA, right ventricle end-systolic area; LASP, left atrial systolic pressure; LADP, left atrial diastolic pressure; mLAP, mean left atrial pressure; PASP, pulmonary arterial systolic pressure; PADP, pulmonary arterial diastolic pressure; mPAP, mean pulmonary arterial pressure; TAPSE, tricuspid annular plane systolic excursion.

### 3.4 Correlation between LAP and echocardiographic parameters of the right heart

**Figure 1:**
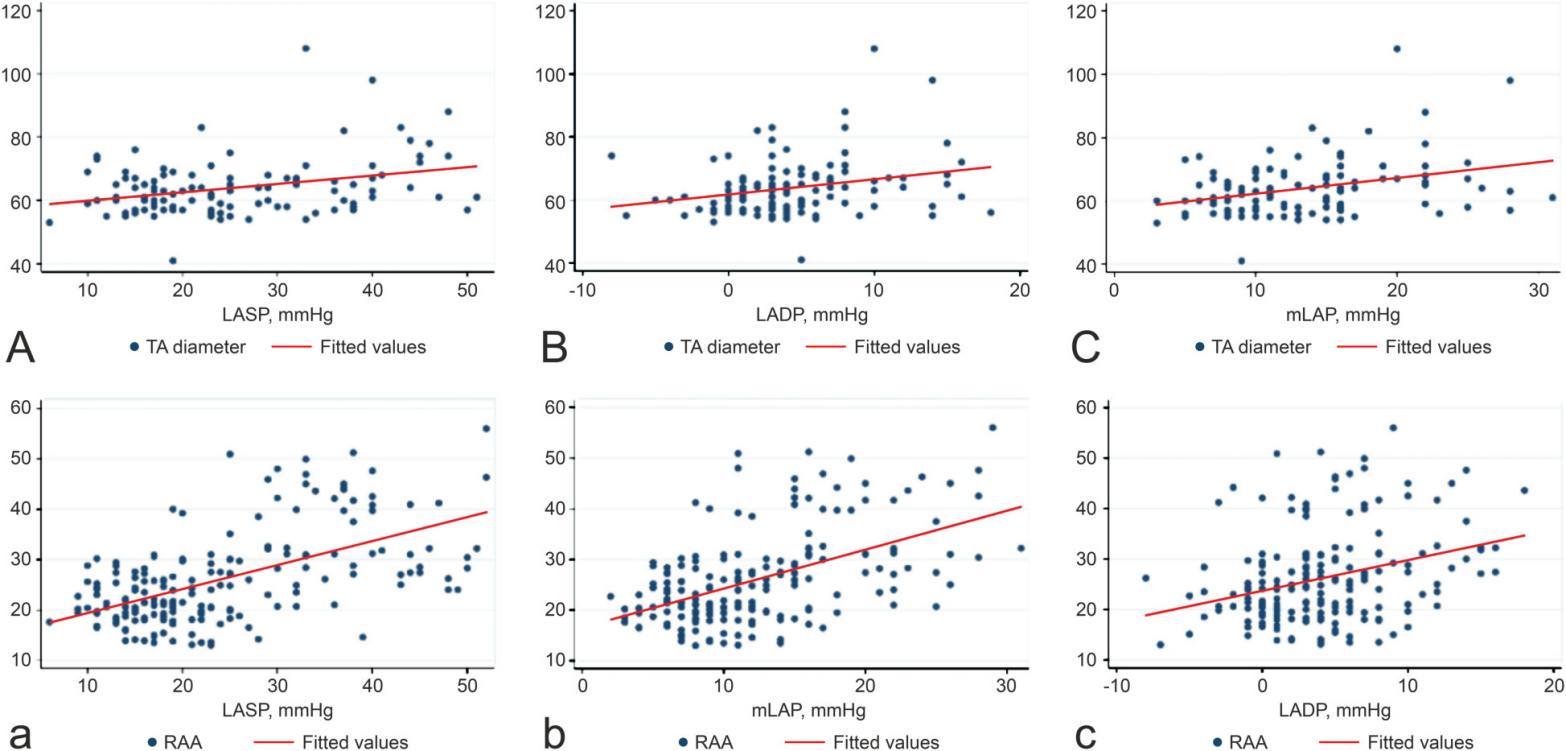
Correlations between LASP, LADP, mLAP, TA **(A–C)**, and RAA **(a–c)**. The unit of measurement for TA is cm, and the units of measurement for RAA is cm. Abbreviations: LASP, left atrial systolic pressure; LADP, left atrial diastolic pressure; mLAP, mean left atrial pressure; TA, tricuspid annulus; RAA, right atrial area.

Table 3 highlights the association between LAP and right heart remodeling in patients with persistent AF. LASP, LADP, mLAP, and LAD showed strong correlations with TR, with correlation coefficients (r) of 0.773, 0.310, 0.640, and 0.212, respectively (*p* < 0.001 for LASP, LADP, and mLAP; *p* = 0.004 for LAD). mLAP was positively associated with TA diameter, RAA, right ventricle end-systolic area, and AF duration but inversely related to TA plane systolic excursion. Figure 1 illustrates the positive correlation between LAP and both TA and RAA. No significant correlation was observed between LADP and AF duration. A strong association was noted between the LAD and RAA dimensions.

**Table 3:** The relationship between parameters.

Patient characteristics	LASP		LADP		mLAP		LAD	
	r	*P*	r	*P*	r	*P*	r	*P*
TASPE, mm	−0.158	0.030	−0.089	0.021	−0.135	0.064	−0.063	0.391
TA diameter, mm	0.360	<0.001	0.185	0.011	0.292	<0.001	0.081	0.272
RAA, cm^2^	0.573	<0.001	0.308	<0.001	0.513	<0.001	0.314	<0.001
RVESA, cm^2^	0.225	0.002	0.150	0.039	0.158	0.030	0.054	0.465
The degree of TR	0.773	<0.001	0.310	<0.001	0.640	<0.001	0.212	0.004
The duration of AF	0.387	<0.001	0.018	0.806	0.190	0.009	0.111	0.133

Abbreviations: TA, tricuspid annulus); RAA, right atrial area); RVESA, right ventricular end-systolic area); TR, tricuspid regurgitation); AF, atrial fibrillation); LASP, left atrial systolic pressure); LADP, left atrial diastolic pressure); mLAP, mean left atrial pressure); LAD, left atrial diameter; TAPSE, tricuspid annular plane systolic excursion).

### 3.5 Univariate and multivariable regression analyses

Table 4 illustrates the outcomes of the univariate and multivariate logistic regression analyses, highlighting the predictors of severe TR. In the univariate analysis, variables such as LAD, LAP, mitral regurgitation, and AF duration (treated as a continuous variable) were significantly associated with TR (all *p* < 0.05). Multivariate analysis revealed that LASP, mitral regurgitation, and AF duration were independent predictors of severe TR after controlling for relevant covariates. Specifically, a 1.53-fold increase in the odds of severe TR was observed for each 1-unit increase in LASP or AF duration. Although mPAP was significant in the univariate analysis, it did not retain significance in the multivariate model.

**Table 4:** Factors influencing tricuspid regurgitation severity: results of univariate and multivariate logistic regression analyses.

Variables	Univariate odds ratio, 95%CI)	*p*-value	Multivariate odds ratio, 95%CI)	*p*-value
LAD, mm	1.08 (1.03,1.14)	0.004	1.00 (0.88,1.14)	0.993
LASP, mmHg	1.36 (1.24,1.49)	<0.001	1.53 (1.18,2.01)	0.002
LADP, mmHg	1.20 (1.10,1.30)	<0.001	1.04 (0.76,1.43)	0.816
mLAP, mmHg	1.39 (1.26,1.53)	<0.001	0.80 (0.52,1.25)	0.327
LVEDs	0.96 (0.89,1.03)	0.221		
LVEDd	0.97 (0.91,1.03)	0.250		
LVEF, %	1.03 (0.97,1.89)	0.306		
Mitral regurgitation	2.77 (1.87,4.12)	<0.001	56.11 (3.30,956.66)	0.005
AF duration, years	1.30 (1.23,1.38)	<0.001	1.53 (1.15,2.04)	0.004
mPAP, mmHg	1.27 (1.18,1.36)	<0.001	1.06 (0.96,1.21)	0.225

Abbreviations: AF, atrial fibrillation; LVEF, left ventricular ejection fraction; LVEDd, left ventricle end-diastolic dimension; LVEDs, left ventricle end-systolic dimension; LAD, left atrial diameter; LASP, left atrial systolic pressure; LADP, left atrial diastolic pressure; mLAP, mean left atrial pressure; mPAP, mean pulmonary arterial pressure.

## 4 Discussion

Our study produced several key findings. First, patients with AF and moderate-to-severe TR had higher LAP and LAA. Second, LAP was significantly associated with the RAA and TA diameter. Notably, this study is the first to identify LASP, mitral regurgitation, and the AF duration as independent predictors of moderate-to-severe TR in patients with persistent AF.

Moderate-to-severe TR was prevalent in patients with persistent AF, with the TR severity correlating with the AF duration (31.7%). Our findings align with those of Patlolla et al., who reported a 38.1% incidence of significant TR 15 years after AF onset (9). The increasing recognition of secondary TR in clinical practice has led to further exploration of its causes. A previous study confirmed that isolated severe TR accounted for 25% of secondary AF cases, and it is often referred to as AF-related TR when pulmonary hypertension or left-sided heart disease is absent (10). More recently, the concept of atrial secondary TR has been introduced to differentiate it from ventricular functional TR. Atrial secondary TR is primarily driven by annular dilation due to atrial enlargement rather than leaflet tethering caused by right ventricular dysfunction (11). Prihadi et al. proposed echocardiographic criteria to distinguish atrial from ventricular functional TR, emphasizing features such as preserved right ventricular function, minimal leaflet tethering, and predominant annular dilation (1). In our cohort, most patients exhibited preserved right ventricular function and minimal leaflet tethering, further supporting the atrial secondary TR mechanism.

In the moderate-to-severe AF group, LA function was closely associated with TR; the left atrium plays a key role in regulating left ventricular filling and overall cardiovascular performance by acting as a reservoir for pulmonary venous flow during ventricular systole, a conduit during early ventricular diastole, and as a booster pump during late ventricular diastole. LA function is typically assessed using the LASP, LADP, and mLAP (12, 13). In our analysis, only LASP remained a significant independent predictor of TR severity in the multivariate model, while LADP and mLAP were not statistically significant. This discrepancy may be explained by the fact that LASP, defined herein as the v-wave peak, is more sensitive to LA stiffness and mitral regurgitation, both of which are associated with pulmonary hypertension and may exert stronger hemodynamic influences on the right heart. In contrast, LADP and mLAP represent baseline or averaged LA filling pressures and may be less effective in capturing dynamic atrial load. In clinical practice, pulmonary capillary wedge pressure (PCWP) is often used as a surrogate for mLAP. Under normal conditions, PCWP closely approximates mLAP, typically exceeding it by 1–3 mmHg. However, this relationship may be confounded by elevated v-waves in mitral regurgitation or by increased pulmonary vascular resistance, both of which impair accurate pressure transmission. In contrast, direct mLAP measurement during LA appendage closure provides a more precise assessment of LA pressure dynamics in such contexts. Persistently elevated LAP may initiate LA remodeling, which exacerbates TR and sustains AF in a vicious cycle. These structural and functional changes in the atrial myocardium—including myofibril depletion, glycogen accumulation, mitochondrial remodeling, and nuclear chromatin dispersion (14)—lead to LA dilation, fibrosis, and impaired contractility, contributing to both diastolic and systolic dysfunction. As atrial compliance declines with advancing AF, biatrial structural changes intensify, perpetuating the burden of functional TR. Importantly, this atrial dysfunction extends to the right heart, promoting RA dilation and TA annular enlargement, which are key contributors to atrial secondary TR. The present findings also support the association between pulmonary hypertension and TR severity, as indicated by the significant univariate correlation between the mPAP and TR grade. However, mPAP was not an independent predictor in the multivariate model, suggesting that pulmonary hypertension in this context is likely a secondary manifestation of chronically elevated left atrial pressure rather than a primary cause of TR. This aligns with the mechanism of atrial functional TR, in which chronic LA pressure overload leads to biatrial remodeling and tricuspid annular dilation without significant pulmonary vascular disease or right ventricular dysfunction. Mitral regurgitation emerged as an independent predictor of TR severity in our multivariate analysis. As a common comorbidity in patients with AF, mitral regurgitation may increase the LAP and volume load, indirectly promoting RA and TA remodeling through pulmonary venous hypertension. Its statistical significance in the multivariate model underscores a potential pathophysiological link between left-sided valvular dysfunction and right-sided chamber changes. Despite the wide confidence interval, the association remains noteworthy and merits further exploration. Recent studies suggest that LA remodeling is not always an irreversible process, and targeted interventions may promote reverse remodeling, potentially improving atrial function and modifying disease progression (15).

Additionally, our results showed that patients with moderate-to-severe TR had lower hemoglobin and hematocrit levels compared to those without significant TR. This may reflect the hemodynamic consequences of venous congestion and volume overload. Chronic hepatic congestion could impair erythropoietin metabolism and iron absorption, leading to reduced hemoglobin synthesis, whereas plasma volume expansion may contribute to hemodilution, further decreasing hematocrit levels. These findings suggest that anemia in patients with moderate-to-severe TR may be multifactorial, and future studies incorporating biomarkers such as NT-proBNP and erythropoietin levels could provide further insights.

Given the progressive nature of TR, early identification of elevated LAP may help guide targeted interventions to prevent TR progression. Aggressive rhythm control strategies or interventions aimed at reducing LAP could mitigate right atrial remodeling and TR severity. Notably, LASP, mitral regurgitation, and AF duration emerged as distinct risk factors for severe TR, further emphasizing the interplay between left and right atrial remodeling in the pathophysiology of TR.

Conversely, LA cardiomyopathy or myocardial injury caused by diabetes, hypertension, or genetic factors can predispose individuals to AF. For instance, Kaivan et al. reported that patients with hypertrophic cardiomyopathy face an elevated risk of AF due to advanced diastolic dysfunction, leading to LA dilatation and remodeling (16, 17). Patients with both hypertension and AF often display distinct echocardiographic features compared with those with hypertension alone. LA strain parameters have been identified as valuable prognostic markers for AF development (18). Additionally, recent evidence has highlighted that persistent LA dysfunction may not only increase the risk of AF recurrence following radiofrequency ablation but also contribute to TR progression through ongoing RA fibrosis and volume overload. Elevated LAP is recognized as an independent predictor of AF recurrence after pulmonary vein isolation (7). These findings collectively reinforce the importance of comprehensive atrial assessment and targeted therapies in patients with AF at risk of TR progression.

### 4.1 Limitations

Our study has some limitations. First, this was a single-center, observational, retrospective, and cross-sectional study, which may have introduced selection bias and limited the ability to establish causality. Longitudinal studies are required to clarify the predictive role of LAP in moderate-to-severe TR. Second, patients were enrolled over an extended period, which may introduce variability in clinical practice and imaging techniques. Third, although patients with paroxysmal AF were excluded, detailed data on the severity of AF burden were not consistently available. Fourth, LA size was assessed using anteroposterior diameter rather than LA volume, and this study lacked dedicated echocardiography specialists for standardized imaging analysis, which may have contributed to interobserver variability. A more comprehensive echocardiographic assessment, including LA volume measurements, would enhance the understanding of its role in TR progression. Finally, TR severity was assessed using the regurgitant jet area on color Doppler imaging, as other quantitative parameters such as coaptation gap or effective regurgitant orifice area (EROA) were not commonly available in the retrospective data. This may limit the accuracy of TR grading compared to contemporary guideline-endorsed multiparametric assessments. Additionally, patients were categorized into two groups (no or mild TR vs. moderate-to-severe TR) due to limited sample size. Although a more granular classification might offer greater resolution, subgrouping was not feasible in the present cohort and should be explored in future studies with larger sample sizes.

## 5 Conclusion

Overall, patients with AF frequently experience significant TR and LA dysfunctions. Elevated LAP, indicative of LA dysfunction, is strongly associated with right heart remodeling and significant TR in patients with persistent AF. Both LASP and AF duration were identified as independent predictors of significant TR.

## Data Availability

The original contributions presented in the study are included in the article/Supplementary Material, further inquiries can be directed to the corresponding author.
